# Shoutai pills improve the quality of oocytes exposed to the chemotherapeutic drug Hydroxyurea

**DOI:** 10.18632/aging.103152

**Published:** 2020-05-10

**Authors:** Yuwei Zhang, Ruizhi Tan, Li Wang, Xiaoyan Shi, Yu Li, Xia Zhong, Xiaoxia He, Bo Xiong

**Affiliations:** 1 Research Center of Combine Traditional Chinese and Western Medicine, Affiliated Traditional Medicine Hospital, Southwest Medical University, Luzhou 646000, Sichuan, China; 2 College of Animal Science and Technology, Nanjing Agricultural University, Nanjing 210095, China; 3 Department of Obstetrics and Gynecology, Affiliated Traditional Medicine Hospital, Southwest Medical University, Luzhou 646000, Sichuan, China

**Keywords:** hydroxyurea, oocyte development, fertilization, shoutai pills, oxidative stress

## Abstract

Hydroxyurea (HU), a DNA synthesis inhibitor, is one of the most common chemotherapeutic drugs that have been widely applied to treat a variety of cancers. HU treatment exhibits severe side effects including renal toxicity, skin toxicity and embryo-toxicity. However, the influence of HU on the female gamete development has not yet fully clarified. Here, we found that HU exposure induced the degeneration of activated follicles after primordial follicle stage, resulting in the depletion of the ovarian reserve. HU exposure also led to the oocyte meiotic maturation arrest via disrupting normal spindle assembly, chromosome alignment and kinetochore-microtubule attachment. Furthermore, exposure to HU impaired the dynamics of ovastacin and Juno, two critical fertilization regulators. Notably, we illustrated that Shoutai pills (STP), a traditional Chinese medicine drug that has been commonly used for the treatment of miscarriage in China, partially restored all of the defects of oocyte development resulting from HU exposure through inhibiting the occurrence of oxidative stress-induced apoptosis. Taken together, our data not only reveal the adverse impact of HU exposure on the female gamete development, but also provide an effective strategy to prevent it, potentially contributing to the improvement of the quality of oocytes from patients treated with HU.

## INTRODUCTION

Hydroxyurea (HU) is a ribonucleotide reductase inhibitor clinically used as an oral antineoplastic for treating several types of cancers including chronic myelogenous leukemia, acute myelogenous leukemia, head and neck cancer, malignant melanoma, ovarian cancer, and polycythemia vera [1]. On the other hand, HU treatment has been reported to cause the myelosuppression, skin toxicity, mild gastrointestinal toxicity, teratogenicity, and abnormalities of renal and liver functions [2–5]. As a potent teratogen, HU treatment causes growth retardation, mortality, and malformations in many experimental species [6–9]. Mechanically, HU inhibits ribonucleotide diphosphatase reductase, the enzyme that catalyzes the reduction of ribonucleotides to the corresponding deoxyribonucleotides that are required for de novo DNA synthesis, and hence leading to the cell death in the embryos [10]. However, inhibition of DNA synthesis cannot fully explain the toxicity induced by HU. The hydroxylamine (-NHOH) group in the HU molecule is able to react with oxygen, producing hydrogen peroxide (H_2_O_2_) that is, in turn, converted to the hydroxyl radical (·OH) [11]. Pretreatment of rabbits with antioxidants (propyl gallate, ethoxyquin, nordihydroguaiaretic acid) or a free radical scavenger (D-mannitol) delays the onset of embryonic cell death and lowers the incidence of malformations caused by HU [12–14], suggesting that the oxidative stress induced by reactive oxygen species (H_2_O_2_, ·OH) contributes to the developmental toxicity of HU. Although various side effects induced by HU treatment have been observed, the adverse impact of HU on the development of female germ cell has not yet been clearly determined.

Shoutai pills (STP), a classical prescription of traditional Chinese medicine that consists of dodder, mistletoe, teasel and gelatin, has been widely used in the treatment of gynecological diseases, especially miscarriage, without any obvious side effects [15, 16]. A recent study documented that STP might improve the endometrial receptivity and promote the embryo implantation by regulating the spatial and temporal balance of Th1/Th2 [17]. Besides, STP could elevate the levels of estrogen and progesterone, ameliorate the endometrial blood flow state, as well as increase the number of blastocyst implantation points, thereby providing a beneficial environment for embryo implantation to increase the success rate of pregnancy [15]. In the meantime, accumulating studies have revealed that STP is able to exert various biological activities via its anti-oxidative, anti-inflammatory and anti-apoptotic effects [18–20].

In the present study, we evaluated the quality of oocytes from HU-administered mice by assessing the critical events and participants during oocyte meiotic maturation, including first polar body extrusion, spindle assembly, chromosome alignment and kinetochore-microtubule attachment. We also tested the dynamics of two key fertilization regulators, ovastacin and Juno. More importantly, we elaborated that STP effectively restored the meiotic failure caused by HU exposure via suppressing oxidative stress-induced apoptosis in oocytes.

## RESULTS

### STP restores the follicle development in HU-administered mice

**Figure 1:**
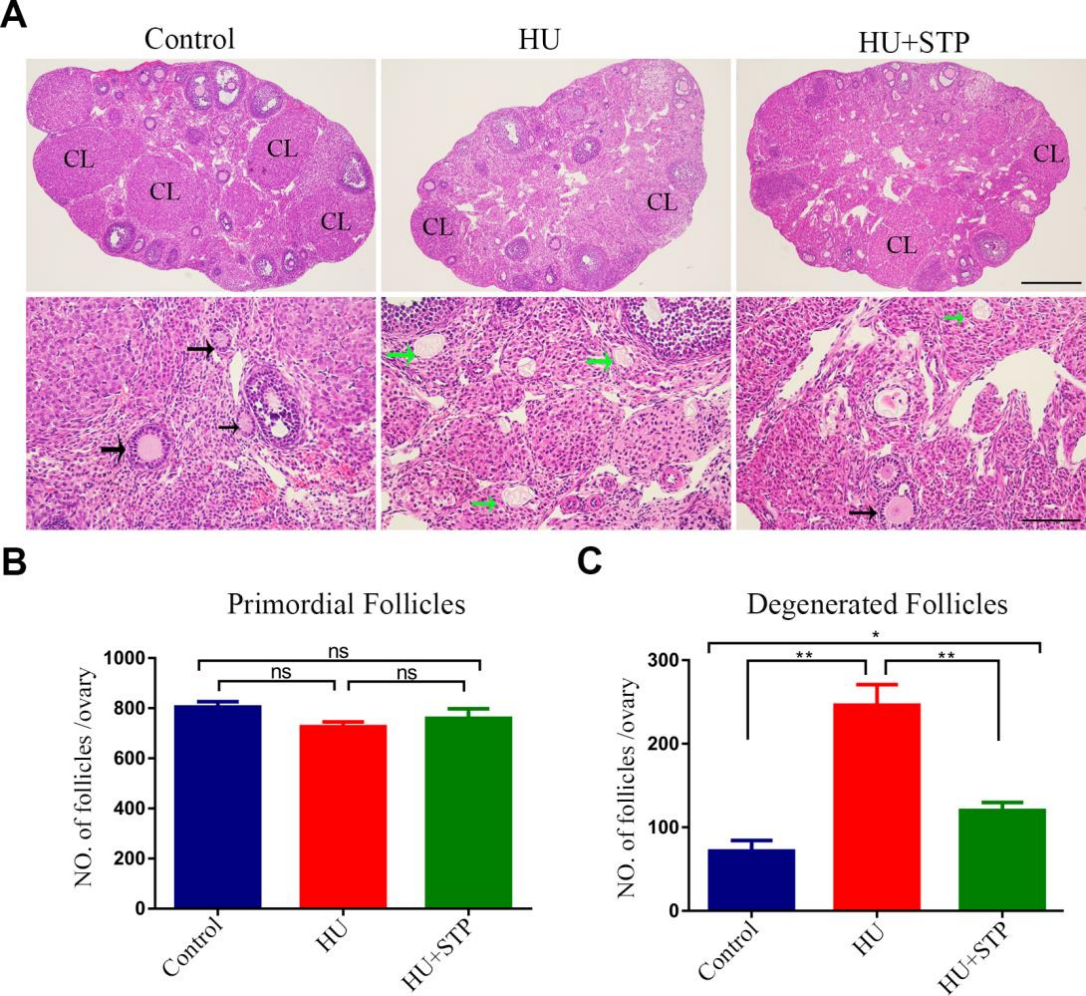
**Effects of STP on the follicle development in HU-exposed ovaries.** (**A**) Histology of ovarian sections in control, HU-exposed and STP-supplemented ovaries. Ovarian sections of 4 μm thickness were prepared and stained with H&E. Black arrows show the growing follicles at different developmental stages; green arrows indicate the developmentally arrested follicles with degenerating oocytes. CL, corpus luteum. Scale bars, 250 μm and 50 μm. (**B**) Quantification analysis of primordial follicles in control, HU-exposed and STP-supplemented ovaries. (**C**) Quantification analysis of degenerated follicles in control, HU-exposed and STP-supplemented ovaries. Data of (**B**, **C**) were presented as mean percentage (mean ± SEM) of at least three independent experiments. *P < 0.05, **P < 0.01.

To examine the changes of ovarian development following HU treatment, we prepared the histological sections of ovaries from mice administered with HU. HE (hematoxylin-eosin) staining analysis revealed that in HU-administered mice a large number of follicular structures were impaired with degenerating oocytes in the transient follicles. Even, some of follicles were lack of oocytes (Figure 1A). By contrast, ovaries from control mice included the growing follicles at different developmental stages and corpora lutea (CL) (Figure 1A). The quantitative analysis showed that the number of primordial follicles was comparable between control and HU-exposed ovaries (Figure 1B). However, a dramatically increased number of developing follicles with developmental arrest and degraded oocytes were present in the HU-exposed ovaries compared to controls (71. 7 ± 12.8, n=6 vs 246 ± 24.9, n=6, *P* < 0.01; Figure 1C). Nevertheless, administration of STP significantly reduced the number of degenerated follicles with the developmental arrest of oocytes induced by HU (120 ± 9.9, n=6, *P* < 0.05; Figure 1C).

### STP promotes the meiotic progression of HU-exposed oocytes

**Figure 2:**
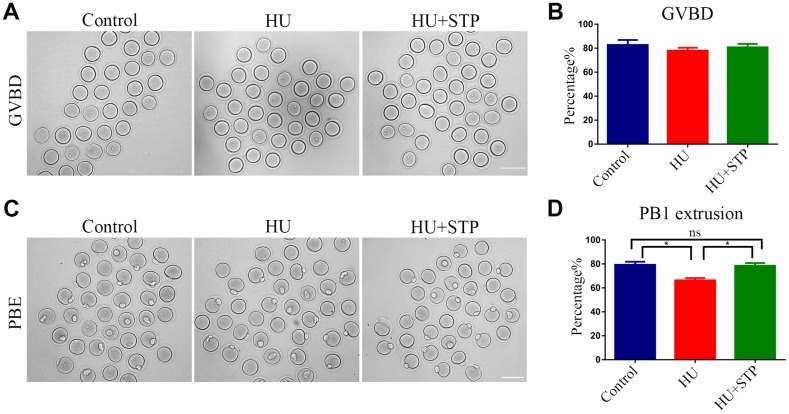
**Effects of STP on the meiotic progression of HU-exposed oocytes.** (**A**) Representative images of oocytes which underwent GVBD (germinal vesicle breakdown) in control, HU-exposed and STP-supplemented groups. Scale bar, 120 μm. (**B**) The rates of GVBD were recorded in control, HU-exposed, and STP-supplemented oocytes. (**C**) Representative images of oocytes which extruded the first polar body (PB1) in control, HU-exposed and STP-supplemented groups. Scale bar, 120 μm. (**D**) The rates of PBE (polar body extrusion) were recorded in control, HU-exposed, and STP-supplemented oocytes. Data of (**B**, **D**) were presented as mean percentage (mean ± SEM) of at least three independent experiments. *P < 0.05.

To ask whether HU exposure would affect oocyte maturation, we observed the meiotic progression of oocytes following HU administration. Germinal vesicle breakdown (GVBD) and polar body extrusion (PBE), two critical developmental events during meiosis, were evaluated. The quantitative analysis showed that HU exposure did not affect GVBD (82.7 ± 4.2%, n=119 vs 78.0 ± 2.4%, n=102; Figure 2A, 2B), but markedly decreased the occurrence of PBE compared to controls (79.3 ± 2.6%, n=105 vs 66.3 ± 1.9%, n=112, *P* < 0.05; Figure 2C, 2D), suggesting that HU exposure causes the meiotic arrest during oocyte maturation. We further tested whether STP has the protective effect against HU-induced meiotic failure, and expectedly found that STP considerably increased the frequency of PBE in HU-exposed oocytes to the control comparable level (78.4 ± 2.3%, n=121, *P* < 0.05; Figure 2C, 2D). Thus, the results indicate that STP is able to relieve the oocyte maturational failure caused by HU exposure.

### STP recovers the spindle defects and chromosome misalignment in HU-exposed oocytes

**Figure 3:**
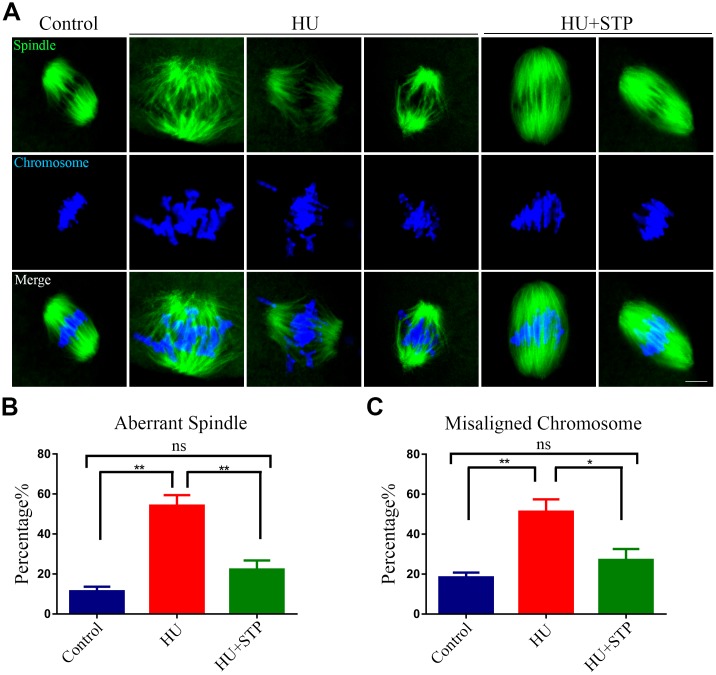
**Effects of STP on the spindle assembly and chromosome alignment in HU-exposed oocytes.** (**A**) Representative images of spindle morphology and chromosome alignment in control, HU-exposed, and STP-supplemented oocytes. M I Oocytes were immunostained with α-tubulin-FITC antibody to visualize the spindles and counterstained with Hoechst to visualize the chromosomes. Scale bar, 2.5 μm. (**B**) The rates of aberrant spindles were recorded in control, HU-exposed, and STP-supplemented oocytes. (**C**) The rates of misaligned chromosomes were recorded in control, HU-exposed, and STP-supplemented oocytes. Data of (**B**, **C**) were presented as mean percentage (mean ± SEM) of at least three independent experiments. *P < 0.05, **P < 0.01.

Given that the arrest of oocyte meiotic progression is always linked with the impairment of spindle structures [21, 22], we examined whether this is the case in HU-exposed oocytes. To this end, oocytes at metaphase I stage were immunolabeled with FITC conjugated α-tubulin- antibody to display the spindle morphologies and counterstained with Hoechst to visualize the chromosome alignment. The results as judged by the immunofluorescence showed that most of control oocytes exhibited a typical barrel-shape spindle apparatus with a well-aligned chromosome at the equatorial plate (Figure 3A). In striking contrast, various morphology-aberrant spindles with misaligned chromosomes were present in HU-exposed oocytes (Figure 3A). Statistically, more than 50% of HU-exposed oocytes displayed the defective spindle/chromosome structure compared to less than 20% in controls (spindle: 11.4 ± 2.2%, n=113 vs 54.2 ± 5.2%, n=109, *P* < 0.01; chromosome: 18.4 ± 2.4%, n=125 vs 51.2 ± 6.2%, n=104, *P* < 0.01; Figure 3B). However, STP administration obviously reduced the abnormal rates caused by HU exposure to a level less than 30% (spindle: 22.2 ± 4.6%, n=98, *P* < 0.01; chromosome: 27.1 ± 5.5%, n=110, *P* < 0.05; Figure 3B, 3C).

### STP maintains the kinetochore-microtubule attachments in HU-exposed oocytes

**Figure 4:**
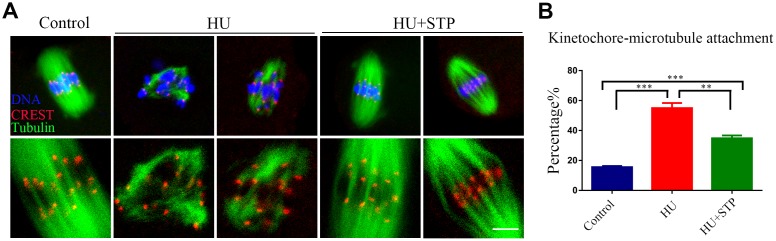
**Effects of STP on the kinetochore-microtubule attachment in HU-exposed oocytes.** (**A**) Representative images of kinetochore-microtubule attachment in control, HU-exposed, and STP-supplemented oocytes. Oocytes were immunostained with α-tubulin-FITC antibody to visualize the spindles, with CREST to display the kinetochores, and counterstained with Hoechst to visualize the chromosomes. Scale bar, 5 μm. (**B**) The rates of defective kinetochore-microtubule attachments were recorded in control, HU-exposed, and STP-supplemented oocytes. Data were presented as mean percentage (mean ± SEM) of at least three independent experiments. **P < 0.01, ***P < 0.001.

Since the defective spindle assembly and incorrect chromosome alignment are usually caused by the improper interaction between kinetochores and microtubules, we thus tested the stability of kinetochore-microtubule attachments following HU exposure. For this purpose, metaphase I oocytes were briefly chilled to induce the depolymerization of microtubules that are not attached to kinetochores and then immunostained with CREST to detect kinetochores, with anti-α-tubulin-FITC antibody to visualize the microtubules and counterstained with Hoechst to observe the chromosomes. It was shown that in most of control oocytes, kinetochores were fully captured by microtubules emanated from the spindle poles (Figure 4A). However, a dramatically increased incidence of free kinetochores without attachment by microtubules was observed in HU-exposed oocytes (Figure 4A). Whereas STP administration significantly reduced the abnormal attachments between kinetochores and microtubules (15.6 ± 0.7%, n=92, *P* < 0.001 vs 55.0 ± 3.4%, n=99 vs 34.8 ± 1.9%, n=105, *P* < 0.01; Figure 4A, 4B).

### STP rescues the abnormal distribution of cortical granules and ovastacin in HU-exposed oocytes

**Figure 5:**
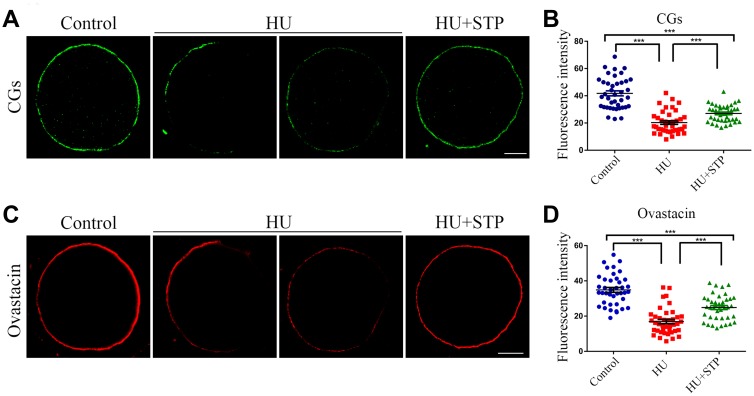
**Effects of STP on the distribution of CGs and ovastacin in HU-exposed oocytes.** (**A**) Representative images of CG dynamics in control, HU-exposed and STP-supplemented oocytes. Scale bar, 20 μm. (**B**) The fluorescence intensity of CGs was measured in control, HU-exposed and STP-supplemented oocytes. (**C**) Representative images of ovastacin dynamics in control, HU-exposed and STP-supplemented oocytes. Scale bar, 20 μm. (**D**) The fluorescence intensity of ovastacin was measured in control, HU-exposed and STP-supplemented oocytes. Data of (**B**, **D**) are presented as mean percentage (mean ± SD) of at least three independent experiments. ***P < 0.001.

Cortical granules (CGs) are oocyte-specific vesicles that localize under the oocyte subcortex to block polyspermy following fertilization. It is noteworthy that the distribution of CGs is usually considered as a critical indicator of oocyte cytoplasmic maturation. Therefore, we examined whether STP would affect the dynamics of CGs by using their marker LCA-FITC. Immunostaining analysis revealed that CGs distributed evenly under subcortical region of oocytes with robust signals in controls, but lost their normal localization pattern in the HU-exposed oocytes by showing the inconsecutive and much weaker signals (Figure 5A). Accordingly, the fluorescent intensity of CG signals was significantly reduced in HU-exposed oocytes compared to controls (41.8 ± 1.8, n=40 vs 20.3 ± 1.5, n=40, *P* < 0.001; Figure 5A, 5B). Conversely, the aberrant dynamics of CGs in HU-exposed oocytes could be rescued by the STP administration (27.1 ± 1.0, n=40, *P* < 0.001; Figure 5A, 5B).

In addition, we also examined the distribution of ovastacin, the first identified component of CGs in mammals that is responsible for the post-fertilization removal of sperm binding site in the zona pellucida to prevent polyspermy. As assessed by the immunofluorescence analysis, anomalous dynamics of ovastacin was found in HU-exposed oocytes by displaying the loss of continuous localization and much decreased intensity of fluorescent signals in comparison with the controls (34.9 ± 1.4, n=40 vs 16.9 ± 1.2, n=40, *P* < 0.001; Figure 5C, 5D). Notably, STP administration reduced the frequency of mis-localization patterns of ovastacin in HU-exposed oocytes (24.9 ± 1.1, n=40, *P* < 0.001; Figure 5C, 5D). Collectively, these observations imply that STP is able to recover the impaired cytoplasmic maturation induced by HU exposure.

### STP restores the protein level of Juno on the membrane in HU-exposed oocytes

**Figure 6:**
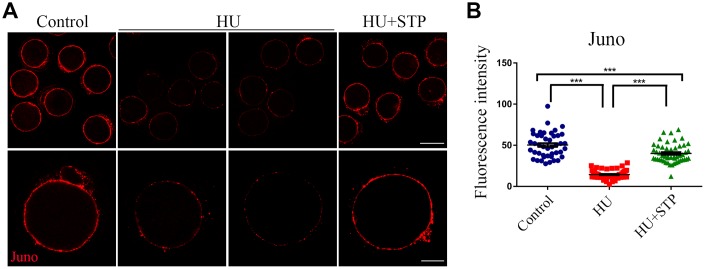
**Effects of STP on the distribution of Juno in HU-exposed oocytes.** (**A**) Representative images of Juno dynamics in control, HU-exposed, and STP-supplemented oocytes. Juno was immunostained with rat monoclonal anti-mouse Folr4 antibody and examined by confocal microscopy. Scale bar, 40 and 20 μm. (**B**) The immunofluorescence intensity of Juno signals was recorded in control, HU-exposed, and STP-supplemented oocytes. Data were presented as mean percentage (mean ± SD) of at least three independent experiments. ***P < 0.001.

Juno is a sperm receptor on the egg membrane which binds to Izumo1 on the sperm head to mediate the sperm-egg fusion and complete the fertilization. Thus, we further investigated the dynamic behavior of Juno upon HU exposure. We performed the immunostaining of Juno and observed that it exhibits a continuous and strong signal on the plasma membrane of control oocytes, whereas became discontinuous distribution pattern with faded signals in HU-exposed oocytes (Figure 6A). This observation was confirmed by the measurement of fluorescence intensity (50.3 ± 2.3, n=44 vs 14.5 ± 0.8, n=50, *P* < 0.001; Figure 6B). In the meantime, STP administration to some extent elevated the intensity of Juno signals impaired by HU exposure (40.1 ± 1.6, n=50, *P* < 0.001; Figure 6B).

### STP decreases ROS levels to suppress DNA damage and early apoptosis in HU-exposed oocytes

**Figure 7:**
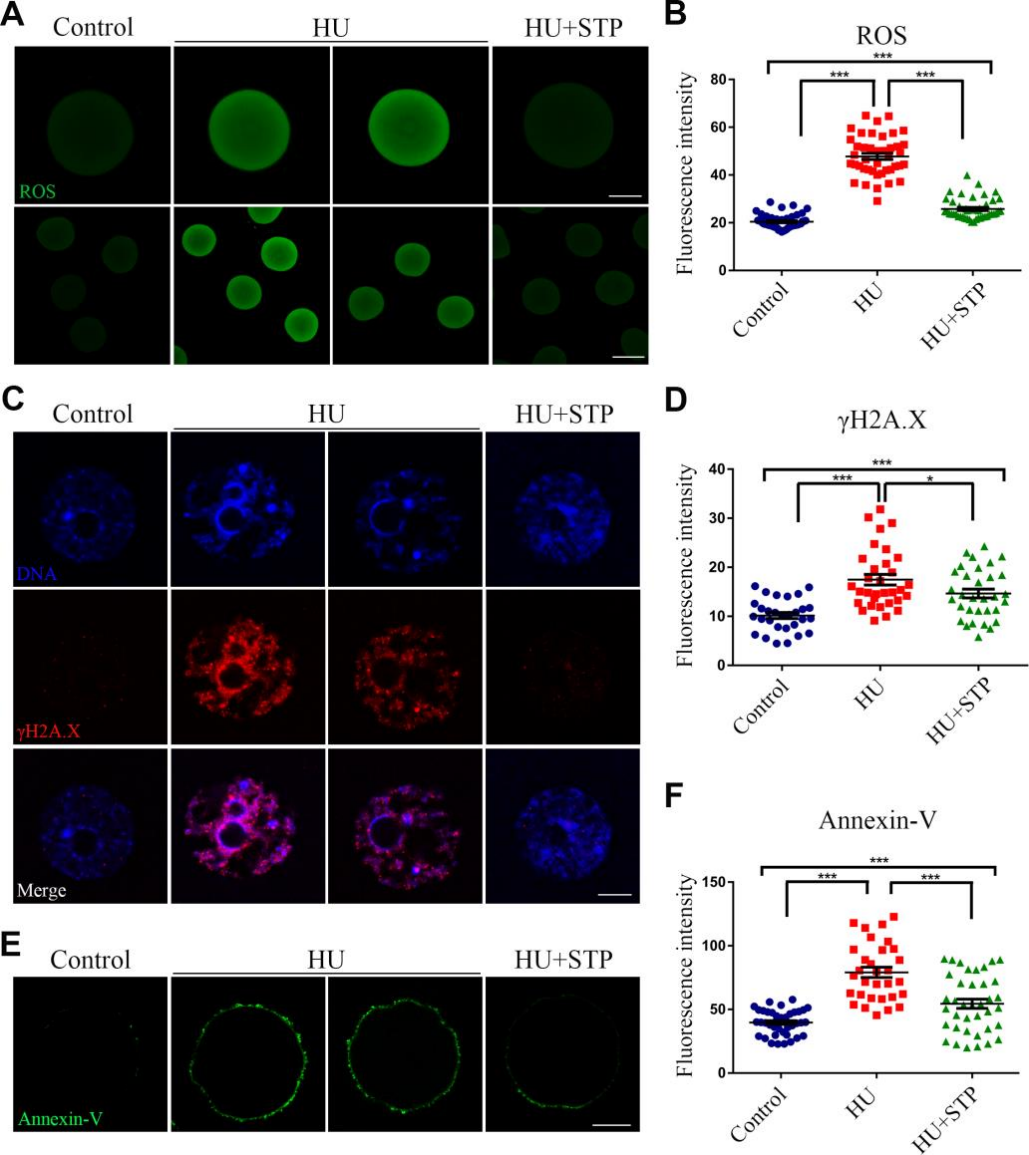
**Effects of STP on the ROS level, DNA damage and early apoptosis in HU-exposed oocytes.** (**A**) Representative images of ROS signals in control, HU-exposed, and STP-supplemented oocytes. Scale bar, 20 and 40 μm. (**B**) The fluorescence intensity of ROS in control, HU-exposed and STP-supplemented oocytes was measured by confocal microscopy using identical settings and parameters. (**C**) Representative images of γH2AX foci in control, HU-exposed, and STP-supplemented oocytes. Scale bar, 5 μm. (**D**) The fluorescence intensity of γH2AX signals was measured in control, HU-exposed, and STP-supplemented oocytes. (**E**) Representative images of apoptotic oocytes in control, HU-exposed, and STP-supplemented oocytes. Scale bar, 20 μm. (**F**) The rates of apoptotic oocytes were recorded in control, HU-exposed, and STP-supplemented oocytes. Data of (**B**), (**D**), and (**F**) were presented as mean percentage (mean ± SD) of at least three independent experiments. *P < 0.05, ***P < 0.001.

Because STP has the anti-oxidative and anti-apoptotic effects on the cellular functions, we hypothesized that STP could inhibit the HU-exposure induced oxidative stress, which accelerates the apoptotic progression of oocytes and thereby leads to the deterioration of critical regulators and events during oocyte meiotic maturation. To test it, we evaluated the ROS levels by DCFH staining. The quantitative analysis of fluorescence intensity showed that ROS signals were considerably higher in HU- exposed oocytes than those in controls (47.8 ± 1.3, n=42 vs 20.4 ± 0.4, n=45, *P* < 0.001; Figure 7A, 7B). Conversely, STP administration weakened the signals of ROS in HU-exposed oocytes (25.8 ± 0.7, n=44, *P* < 0.001; Figure 7A, 7B).

Since the oxidative stress always induces the accumulation of DNA damage and occurrence of apoptosis [23, 24], we further confirmed if this is the case in HU-exposed oocytes. The level of DNA damage was assessed by the immunostaining of γH2AX foci and determined by the measurement of fluorescence intensity. The results revealed that DNA damage level was remarkably elevated in HU-exposed oocytes in comparison with the controls, whereas STP administration significantly decreased the abnormalities (10.2 ± 0.6, n=29, *P* < 0.001 vs 17.5 ± 1.1, n=32 vs 14.6 ± 0.9, n=32, *P* < 0.05; Figure 7C, 7D).

Next, we examined the apoptotic status of oocytes by Annexin-V staining. We observed that the fluorescent signals were barely observed in control oocytes, but apparently present on the membrane of HU-exposed oocytes, indicative of early apoptosis (Figure 7E, 7F). Accordingly, the occurrence of apoptosis was dramatically higher in HU-exposed oocyte than that in controls, but reduced in STP-administered oocytes (39.7 ± 1.5, n=40, *P* < 0.001 vs 79.1 ± 4.0, n=31 vs 54.5 ± 3.6, n=38, *P* < 0.001; Figure 7E, 7F).

## DISCUSSION

Hydroxyurea, a DNA synthesis inhibitor, is one of the most common chemotherapeutic drugs that have been widely used in the treatment of inoperable carcinoma of the ovary [25, 26]. HU treatment exhibits a variety of severe side effects including renal toxicity, skin toxicity and embryo-toxicity [27]. Treatment of women of reproductive age with HU may lead to loss of primordial follicles, resulting in the depletion of the ovarian reserve and consequently premature ovarian failure [10]. However, the impact of HU exposure on the oocyte quality has not fully determined.

To investigate it, we firstly observed the follicular development by ovarian section following HU exposure, and found that a higher frequency of degenerated follicles without oocytes were present in HU-administered mice, which is consistent with the previous report that HU treatment might result in the loss of ovarian reserve. We next monitored the oocyte meiotic progression, a key indicator of oocyte maturation and development. Our findings revealed that HU exposure caused oocyte maturation arrest by displaying the failure of polar body extrusion. This was due to the occurrence of defective spindle assembly and chromosome alignment. Because spindle/chromosome abnormalities are always caused by the error of kinetochore-microtubule attachment [28, 29], we further assessed it to show that a large number of free kinetochores which were not attached by the microtubules were present in HU-exposed oocytes, leading to misaligned chromosomes. Therefore, these observations suggest that the nuclear maturation of oocytes is impaired upon HU exposure.

Mammalian cortical granules are oocyte-specific and membrane-bound vesicles that form a uniform layer in the subcortical region of fully grown oocytes to exert functions during fertilization and prevention of polyspermy [30]. Meanwhile, the normal distribution of CGs is often regarded as a sign of oocyte cytoplasmic maturation. We then tested if HU exposure would perturb the dynamics of cortical granules. Our data illustrated that cortical granules lost its normal distribution pattern in the subcortex of HU-exposed oocytes, indicating that cytoplasmic maturation is impaired as a result of HU exposure. Ovastacin, a critical fertilization regulator and component of cortical granules, is responsible for post-fertilization cleavage of ZP2 (zona pellucida protein 2) at the N-terminus, the sperm binding site in the zona pellucida, to definitively block polyspermy [30]. However, if it is exocytosed out of oocytes prior to fertilization, the sperm binding site will be prematurely removed, leading to the failure of sperm binding and fertilization. In line with the above observation of cortical granule dynamics, we found that the localization of ovastacin was disrupted and the amount was reduced in HU-exposed oocytes, implying that sperm binding ability of oocytes following HU exposure might be compromised. In addition, we also examined the behavior of another key fertilization regulator that mediates the sperm-oocyte fusion, Juno, on the oocyte membrane, and discovered that HU exposure remarkably impaired the distribution dynamics of Juno, indicating that sperm-oocyte fusion might be damaged. Altogether, these observations suggest that cytoplasmic maturation of oocytes is perturbed upon HU exposure.

Consistent with the previous study showing that HU exposure induces the production of oxidative stress in cells [31–33], our findings revealed that HU exposure generated higher levels of ROS and DNA damage, finally resulting in the occurrence of apoptosis. STP, due to its strong antioxidant capacity, effectively restored HU-exposure caused meiotic failure of oocytes via eliminating the excessive accumulated ROS. In human studies, the use of 25 g STP has been demonstrated to improve the quality of oocytes and embryos as well as the pregnancy outcomes without any obvious physiologic side effects [15, 34, 35]. In our study, the dose of 3.6 g/kg body weight of STP used for mice is equal to 20 g for human use according to the dose conversion between mice and humans. Therefore, our findings give an implication that STP has a potential role in preventing the deterioration of oocytes exposed to HU when supplied concurrently, but whether it can reverse the adverse effects of HU that have already accumulated in oocytes needs the future investigations.

Collectively, we provide a body of evidence documenting that STP ameliorates the quality of oocytes exposed to HU by promoting both nuclear and cytoplasmic maturation through inhibiting HU-induced oxidative stress. Thus, our findings give an implication that the combination of STP with chemotherapeutic drug HU could reduce the chemotherapy-associated reproductive toxicity.

## MATERIALS AND METHODS

### Animals and feeding treatment

All experiments were approved by the Animal Care and Use Committee of Southwest Medical University and Nanjing Agricultural University, China, and were performed in accordance with institutional guidelines. The ICR mice were kept at controlled condition of temperature (23 ± 2°C) and illumination (12 hours light-dark cycle), and had free access to food and water throughout the period of the study. Female mice were used for oocyte collection.

### Oocyte collection and culture

Fully-grown oocytes arrested at prophase of meiosis I were collected from ovaries in M2 medium (Sigma, St Louis, MO, USA). Only those immature oocytes displaying a germinal vesicle (GV) were cultured further in M16 medium (Sigma, St Louis, MO, USA) under liquid paraffin oil at 37°C in an atmosphere of 5% CO_2_ incubator for *in vitro* maturation. At different time points after culture, oocytes were collected for subsequent analysis.

### HU and STP treatment

Female ICR mice (6-week-old) were randomly assigned to three groups: a control group, a HU-exposed group, and a “HU+STP” group. On average, mice received HU of daily oral doses of 500 mg/kg body weight and/or STP of daily oral doses of 3.6 g/kg body weight in PBS carrier at 10:00 am everyday for 7 days preceding oocyte collection and analysis. The data analysts were blind to the experimental treatments.

### Immunofluorescence and confocal microscopy

Mouse oocytes were fixed in 4% paraformaldehyde in PBS (pH 7.4) for 30 minutes and permeabilized in 0.5% Triton X-100 for 20 minutes at room temperature. Then, oocytes were blocked with 1% BSA-supplemented PBS for 1 hour and incubated at 4°C overnight or at room temperature for 4 hours with anti-α-tubulin-FITC antibody (1:200, Sigma, USA), anti-centromere CREST antibody (1:50, Antibodies Incorporated, CA, USA), rat monoclonal anti-mouse Folr4-FITC antibody (1:100, BioLegend, CA, USA), or rabbit polyclonal anti-mouse ovastacin antibody (1:100, obtained from Dr. Jurrien Dean). After washing four times (5 minutes each) in PBS containing 1% Tween-20 and 0.01% Triton X-100, oocytes were incubated with an appropriate secondary antibody for 1 hours at room temperature. After washing three times, oocytes were counterstained with Hoechst 33342 (10 μg/ml) for 10 minutes. Finally, oocytes were mounted on glass slides and viewed under a confocal laser scanning microscope (Carl Zeiss LSM 700 META).

### Determination of ROS generation

To determine the levels of intracellular ROS production, oocytes were incubated with the oxidation-sensitive florescent probe dichlorodihydrofluorescein (DCFH) for 30 minutes at 37°C in D-PBS containing 10 μmol/L DCFH diacetate (DCFH-DA) (Beyotime Institute of Biotechnology, China). Then, oocytes were washed three times in D-PBS containing 0.1% BSA and then placed on glass slides. The measurement of the fluorescent intensity in each oocyte was carried out by a Zeiss LSM 700 META confocal system with the same scanning settings.

### Annexin-V staining

According to the manufacturer’s instruction (Beyotime Institute of Biotechnology, Hangzhou, China), mouse oocytes were labeled with Annexin-V staining kit. After washing twice in PBS, the live oocytes were stained for 30 minutes in the dark with 90 μL of binding buffer containing 10 μL of Annexin-V-FITC. Fluorescent signals were measured by the confocal microscope with the same scanning settings.

### Statistical analysis

All percentages from at least three repeated experiments were expressed as mean ± SEM or mean ± SD, and the number of oocytes observed was labeled in parentheses as (n). Data were analyzed by paired-samples t-test, which was provided by SPSS 16 statistical software. The level of significance was accepted as P < 0.05.
